# Growth of Community Outpatient Care in the Veterans Affairs System After the MISSION Act

**DOI:** 10.1007/s11606-024-08787-7

**Published:** 2024-05-09

**Authors:** Jean Yoon, Kritee Gujral, Clara Dismuke-Greer, Jennifer Y. Scott, Hao Jiang

**Affiliations:** 1 Health Economics Resource Center (HERC), VA Palo Alto Health Care System, Menlo Park, CA USA; 2 UCSF School of Medicine, Department of General Internal Medicine, San Francisco, CA USA; 3 Department of Psychiatry and Behavioral Sciences, Stanford University School of Medicine, Stanford, CA USA; 4 Center to Improve Veteran Involvement in Care, VA Portland Health Care System, Portland, OR USA

**Keywords:** community care, MISSION, primary care, emergency care, veteran

## Abstract

**Background:**

The Maintaining Internal Systems and Strengthening Integrated Outside Networks (MISSION) Act of 2018 authorized a major expansion of purchased care in the community for Veterans experiencing access barriers in the Veterans Affairs (VA) health care system.

**Objective:**

To estimate changes in primary care, mental health, and emergency/urgent care visits in the VA and community fiscal years (FY) 2018–2021 and differences between rural and urban clinics.

**Design:**

A national, longitudinal study of VA clinics and outpatient utilization. Clinic-level analysis was conducted to estimate changes in number and proportion of clinic visits provided in the community associated with the MISSION Act adjusting for clinic characteristics and underlying time trends.

**Participants:**

In total, 1050 VA clinics and 6.6 million Veterans assigned to primary care.

**Main Measures:**

Number of primary care, mental health, and emergency/urgent care visits provided in the VA and community and the proportion provided in the community.

**Key Results:**

Nationally, community primary care visits increased by 107% (50,611 to 104,923), community mental health visits increased by 167% (100,701 to 268,976), and community emergency/urgent care visits increased by 129% (142,262 to 325,407) from the first quarter of 2018 to last quarter of 2021. In adjusted analysis, after MISSION Act implementation, there was an increase in community visits as a proportion of total clinic visits for emergency/urgent care and mental health but not primary care. Rural clinics had larger increases in the proportion of community visits for primary care and emergency/urgent care than urban clinics.

**Conclusions:**

After the MISSION Act, more outpatient care shifted to the community for emergency/urgent care and mental health care but not primary care. Community care utilization increased more in rural compared to urban clinics for primary care and emergency/urgent care. These findings highlight the challenges and importance of maintaining provider networks in rural areas to ensure access to care.

**Supplementary Information:**

The online version contains supplementary material available at 10.1007/s11606-024-08787-7.

## INTRODUCTION

The Veterans Affairs (VA) health care system cares for more than 9 million Veterans through its national integrated delivery system of outpatient clinics and hospitals. Care is purchased from community providers when services are not available at a VA site or certain access standards cannot be met. Major changes occurred under the Veterans Access, Choice and Accountability Act (Choice Act) of 2014 which authorized an expansion to VA-purchased community care to address wait time and drive time barriers to obtaining VA care. This policy led to an increase in outpatient care provided in the community.^1–3^

Subsequently, the Maintaining Internal Systems and Strengthening Integrated Outside Networks (MISSION) Act of 2018 further expanded the purchase of care in the community and broadened eligibility.^4^ Little is known how the MISSION Act affected utilization of community outpatient services, especially when the COVID-19 pandemic markedly reduced health care utilization for many services.^5^ While recent studies found large increases in community care for emergency care, urgent care, and colonoscopies following the MISSION Act,^6–8^ only one formally evaluated the impact of the MISSION Act on colonoscopies. No prior studies evaluated the impact of the MISSION Act broadly for primary care, mental health, and emergency/urgent care.

Similar to the general population, Veterans in rural areas are more likely to face provider shortages and long travel times to care.^9,10^ Since there are fewer VA clinics and medical centers in rural areas, Veterans in rural areas historically used more community care than Veterans in urban areas.^11,12^ However, some services may be less available in rural areas, and uptake of some community services may be lower in rural compared to urban areas. Examining potential rural–urban differences in the growth of different types of community outpatient care is critical for understanding the impact of the MISSION Act in addressing access barriers and planning for care coordination across VA and non-VA care. In this study, we examined overall changes in VA foundational services for primary care, mental health, and emergency/urgent care visits in the VA and community before and after the MISSION Act during fiscal years (FY) 2018–2021 while estimating differences by rurality.

## METHODS

We conducted a national, longitudinal study of VA primary care clinics and quarterly utilization of Veterans assigned to a primary care team in these clinics and had outpatient utilization in at least one quarter FY 2018–2021. The FY begins on October 1 and ends on September 30 of the following calendar year. Our study period includes the periods pre- and post-MISSION Act implementation and pre- and post-COVID-19 emergency response in the VA when elective services were deferred and many services transitioned to virtual modalities.^13^ All utilization data were obtained from the VA Corporate Data Warehouse. We measured clinic characteristics from VA administrative data. This analysis was conducted as part of a larger evaluation on VA primary care and mental health care models for the purposes of quality improvement.^7^

### Cohort and Data Sources

We included 1050 VA outpatient clinics identified as providing primary care through the VA Site Tracking (VAST) database from fiscal years (FY) 2018–2021. These clinics included those based in VA medical centers and community-based outpatient clinics. We excluded clinics with fewer than 500 patients per quarter assigned to primary care as many of them were mobile sites with low visit volumes. We measured utilization for Veterans assigned to a primary care team in these clinics in the VA Re-engineered Primary Care Management Module in at least one quarter between FY 2018 and 2021. We excluded Veterans who did not have any VA or community primary care, mental health, or emergency/urgent care throughout the study period (*N* = 504,250). Our analysis included a total of 16,800 clinic-quarter observations.

### Study Measures

Primary care, mental health, and emergency/urgent care visits were measured from VA outpatient records and community care claims data and included both in-person and virtual modalities. VA Primary care, emergency/urgent care, and mental health care were categorized based on VA clinic stop codes. Veterans’ community outpatient visits were obtained from several sources of community care claims: Fee Basis, Program Integrity Tool, Community Care Reimbursement System, and the Electronic Claims Adjudication Management System. Primary care, emergency/urgent care, and mental health care were categorized based on an algorithm using procedure codes and provider classification codes originally developed for Medicare data and adapted for community care claims records.^14,15^ For patients assigned to each clinic, we estimated the clinic proportion of community visits as the number of visits obtained in the community divided by the total number of visits obtained in the VA and community for each type of care in each FY quarter.

Our analyses adjusted for clinic characteristics that were obtained from the VA Site Tracking (VAST) Database. Clinic type was categorized as medical center-based clinic, primary care community-based outpatient clinic, multi-specialty community-based outpatient clinic, or other/unknown type. Geographic region was categorized as Northeast, Midwest, Southeast, Gulfstream, or West. Rural or urban location was categorized based on rural–urban continuum codes from the Department of Agriculture.^10^ There were 21 clinics that did not have a rural/urban designation, so we assigned clinics as rural if more than 50% of their patients lived in a rural area; the others were categorized as urban.

For descriptive purposes, we also measured the mean of patients’ distance to their closest VA site for primary and secondary care as reported in the VA Geospatial Services Support Center Files. We compared the mean of patients’ distance to their closest VA site for those assigned to primary care in rural and urban clinics.

### Analytic Methods

We first conducted unadjusted analysis to summarize total visits and patients nationally in each quarter, the mean number of VA + community visits per patient by type of care, and the difference in total number of VA and community care visits and patients over the 4-year period. We also estimated the mean number of community visits and the mean proportion of community care visits by type of care in all clinics in each FY quarter. In adjusted analyses, we conducted regression models using primary care clinic in each FY quarter as the units of analysis. Separate fractional probit regression models were used to estimate the adjusted proportion of community visits for primary care, mental health, and emergency/urgent care. Models included an indicator for each FY quarter to adjust for common shocks in each FY quarter including the effects of the COVID-19 pandemic, a rural/urban indicator with a separate interaction term between rural/urban and FY quarter, indicators for clinic type, and indicators for geographic region. Standard errors were adjusted for clustering within clinic.^11^ We estimated marginal effects for each FY quarter in rural and urban clinics to obtain the mean clinic proportion of community care visits and their 95% confidence intervals.

In order to estimate the association between the MISSION Act and community care utilization by clinics, we conducted four separate Wald tests to detect significant differences in the mean coefficients for the FY quarter between the pre-MISSION Act period (FY 2018 quarter 1–FY 2019 quarter 3 since the MISSION Act was implemented on June 6, 2019, at the end of this period) and (1) the period immediately after the MISSION Act (FY 2019 quarter 4–FY 2020 quarter 2), (2) the period immediately after the COVID-19 emergency response (FY 2020 quarter 3–FY 2021 quarter 1), (3) the period following distribution of the COVID-19 vaccines (FY 2021 quarter 2–FY 2021 quarter 4), and (4) the entire post-MISSION Act period (FY 2019 quarter 4–FY 2021 quarter 4).

We also conducted separate regression models to estimate the mean clinic number of community care visits in each FY quarter to examine changes in total community care visits over time. Regression models were similar to models that estimate proportion of community care visits except that linear models were used.

In sensitivity analyses, we used clinic fixed effects to examine changes in the mean number of clinic visits and proportion of outpatient visits for community care over the study period. We tested differences in each time period using Wald tests similar to the original models. All analyses were conducted in Stata 18 from 9/13/2023 to 3/19/2024.

## RESULTS

### Change in Total Visits and Patients FY 2018–2021

There was a total of 1050 VA clinics with the number of Veterans receiving outpatient care each quarter increasing from 3.1 million to 3.2 million from beginning of FY 2018 to end of FY 2021 and a total of 6.6 million unique patients during the study period. Clinics were evenly distributed across geographic regions with the most common type being primary care community-based outpatient clinic and 38% of clinics located in rural areas (Table 1). Mean patient age was 61 years, and 98% of patients were male. Clinics had a mean number of 3009 (SD = 3367) patients, 4858 (SD = 5472) primary care visits, 2858 (SD = 3846) mental health visits, and 701 (SD = 1072) emergency/urgent care visits each quarter. Patients in rural clinics traveled much farther to reach their closest VA primary and secondary care sites than patients in urban clinics (Table 2).

**Table 1:** VA Primary Care Clinic Characteristics, *N* = 1050

Clinic characteristics	*N* (%)
Region	
East	252 (24%)
Southeast	177 (17%)
Gulfstream	189 (18%)
Midwest	252 (24%)
West	179 (17%)
Clinic type	
VA medical center	168(16%)
Primary care community-based outpatient clinic	515 (49%)
Multi-specialty community-based outpatient clinic	199 (19%)
Other type	168 (16%)
Location	
Rural	399 (38%)
Urban	651 (62%)
Patient demographics	
Mean patient age	61 (5)
Mean percent male patients	92% (6)
Mean percent marrried patients	57% (9)
Mean percent unmaried patients	41% (8)
Mean percent unknown marital status	2% (2)
Mean percent White Patients	70% (20)
Mean percent Black Patients	12% (16)
Mean percent Latino Patients	5% (10)
Mean percent other race patients	12% (9)
Mean percent VA priority groups 1–2	40% (10)
Mean percent VA priority groups 3–4	14% (0.04)
Mean percent VA priority groups 5–6	21% (5)
Mean percent VA priority groups 7–8	18% (7)
Mean percent uknown VA priority group	7% (5)
Mean Elixhauser comorbidity score	1.3 (0.3)
Mean number of patients/quarter	3009 (3367)
Mean number of VA primary care visits/quarter	4858 (5472)
Mean number of VA mental health visits/quarter	2858 (3846)
Mean number of VA emergency/urgent care visits/quarter	701 (1072)

**Table 2:** Patients’ Mean Distance to Primary Care Clinics, *N* = 1050

Clinic location	VA primary care mean distance in miles (SD)	VA secondary care mean distance in miles (SD)
Rural, *N* = 399	28 (22)	90 (49)
Urban, *N* = 651	14 (5)	44 (34)

**Figure 1:**
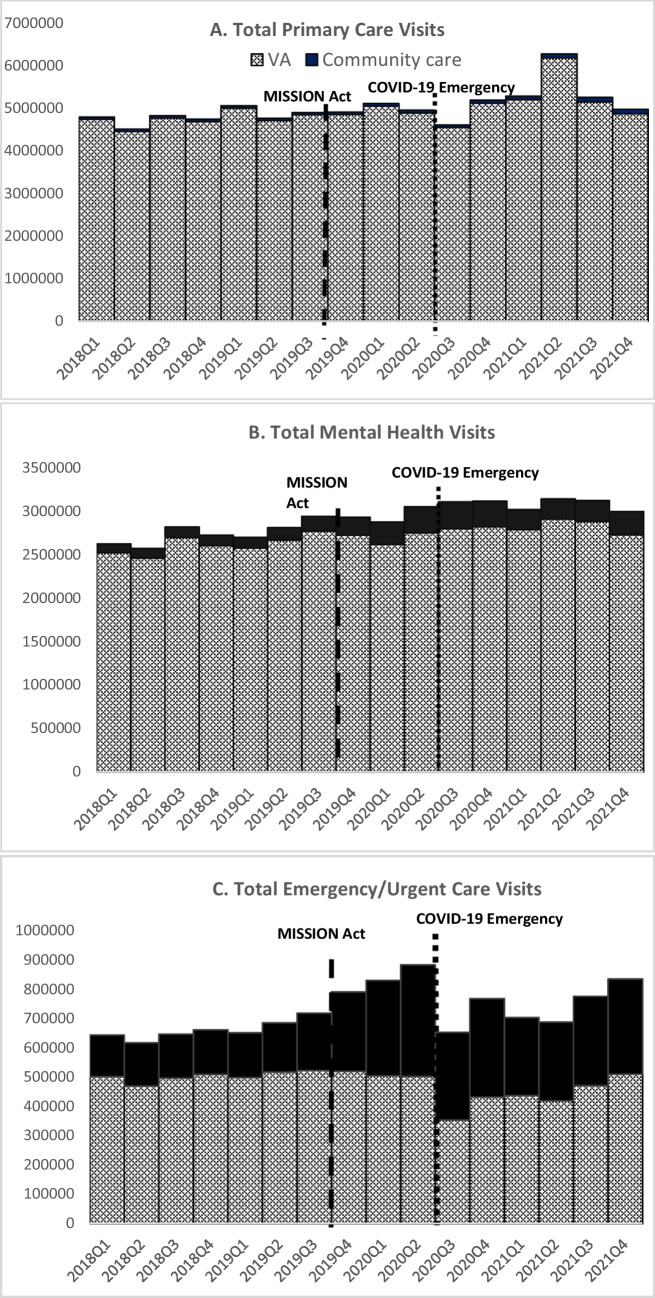
Total unadjusted number of outpatient visits for primary care, mental health, and emergency/urgent care in each Fiscal Year-quarter, Fiscal Years 2018–2021. The MISSION Act began implementation on June 6, 2019. VA COVID-19 pandemic national emergency response began on March 15, 2020.

Nationally, both VA and community outpatient visits increased over the 4-year period. Community primary care visits increased 107% (50,611 to 104,923) versus 3% for VA (4,742,429 to 4,872,344), community mental health visits increased 167% (100,701 to 268,976) versus 8% for VA (2,526,999 to 2,731,276), and community emergency/urgent care visits increased 129% (142,262 to 325,407) versus 2% for VA (502,544 to 510,656) from the first quarter of 2018 to last quarter of 2021 (Fig. 1). The mean number of visits per patient for all types of care remained similar over time (Appendix Figure A1).

Following the COVID-19 pandemic emergency response, total VA and community primary care visits were reduced in the third quarter of FY 2020 while emergency/urgent care visits were reduced for most of the third quarter-FY 2020 through the end of FY 2021. In contrast, VA and community mental health visits increased in the two quarters following the COVID-19 pandemic response. At the end of FY 2021, community visits comprised 2% of all primary care visits (104,923 of 4,977,267), 9% of all mental health visits (100,701 of 3,000,252), and 39% of all emergency/urgent care visits (325,407 of 836,063).

### Clinic-Level Visits and Proportion of Community Care Visits

After adjusting for clinic characteristics, the mean number of primary care visits in the community increased in both rural and urban clinics from beginning to end of the study period but was higher in urban clinics compared to rural clinics (Appendix Figure A3). The mean number of mental health visits in the community increased over time in urban clinics but not rural clinics while both rural and urban clinics had an increase in mean number of emergency/urgent care visits in the community over time. All types of care had short-term decreases in the mean numbers of visits at the beginning of the COVID-19 pandemic. All types of care had a significant increase in total community visits after the MISSION Act (*P* < 0.001; Appendix Table A1).

**Figure 2:**
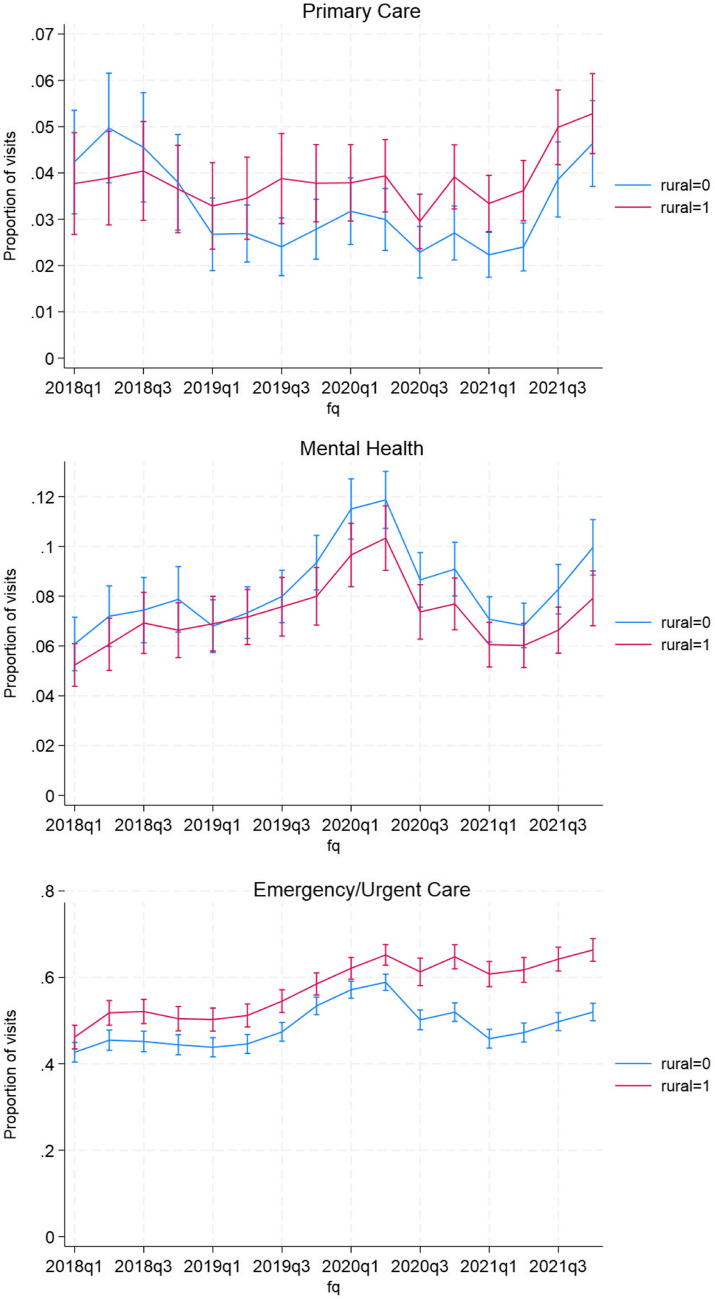
Adjusted proportion of clinic visits provided in the community for rural and urban clinics, Fiscal Years 2018–2021

There were very similar patterns in the proportion of outpatient visits in the community over the study period in both unadjusted (Appendix Figure A2) and adjusted analyses (Fig. 2). In adjusted analyses estimating proportion of outpatient visits in the community, the mean proportion for primary care was slightly lower in FY 2019–2020 compared to the beginning of FY 2018, with no consistent difference between rural and urban clinics. There was also no significant difference in mean adjusted proportion of primary care community visits after the MISSION Act (*P* = 0.116; Appendix Table A2). In contrast, there was an increasing trend in adjusted proportion of mental health visits in the community between FY 2018 and FY 2020 and a significant increase in the adjusted proportion in the community after the MISSION Act (*P* < 0.001). This increase occurred in both rural and urban clinics although there was a temporary decrease in the proportion after the COVID-19 emergency response (mid-FY 2020 to mid-FY 2021). For emergency/urgent care visits, there was a significant increase in the proportion of visits in the community after the MISSION Act (*P* < 0.001) for rural and urban clinics. Rural clinics had a significantly higher proportion of visits in the community compared to urban clinics.

In sensitivity analyses, we used a clinic fixed effect instead of regional effects, and we found similar results with respect to differences in mean clinic visits and proportion of outpatient visits in the community pre- and post-MISSION Act.

## DISCUSSION

This is the first study to examine changes in different types of VA and community outpatient care more than 1 year prior and 2 years after the MISSION Act. From FY 2018 to FY 2021, community visits for primary care, mental health care, and emergency/urgent care increased 107%, 167%, and 129%, compared to VA visits increasing only 3%, 8%, and 2%, respectively. The MISSION Act was associated with a larger proportion of care provided in the community for emergency/urgent care and mental health with almost two out of every five emergency/urgent care visits and one out of every nine mental health visits provided in the community at the end of 2021. Primary care was less commonly provided by community providers although community primary care visits increased over the 4-year period.

Rural clinics had a larger increase in the proportion of care provided in the community for emergency/urgent care than urban clinics following the MISSION Act. Veterans frequently travel farther to obtain VA-provided emergency/urgent care and specialty mental health services from large VA medical centers and multi-specialty clinics than for primary care which is provided through those same sites as well as many VA community-based outpatient clinics.^16^ Hence, Veterans appeared more likely to shift their utilization of emergency/urgent care and specialty mental health care to the community after access to purchased care expanded.

The use of purchased care in the community was designed to reduce wait time and drive time barriers to care, and Veterans in rural areas, who typically face these barriers to VA care, consequently accessed more community care.^12,13^ Community providers may play a critical role in providing timely care for rural Veterans for emergency conditions, although it is unknown whether greater use of community emergency/urgent care led to improved outcomes for Veterans in rural areas. These results highlight the need for broader provider networks in rural areas where the relative supply of providers is much lower than in urban areas.

While the proportion of care provided in the community for mental health and emergency/urgent care grew after the MISSION Act, the growth in community care appeared to be temporarily reduced due to the COVID-19 pandemic. The pandemic may have caused disruptions to care leading to fewer referrals to community. However, community care continued its steady growth as the health care system recovered from the pandemic. We also found a drop in total utilization for primary care and emergency/urgent care at the beginning of the COVID-19 pandemic with emergency/urgent care reduced for a longer period of time. The long-term impacts of this reduction in utilization and potential deferral of care have yet to be determined.^15^ Total mental health visits increased slightly after the pandemic began which has also been noted outside the VA.^16^

### Limitations

Study limitations include the inability to draw causal inferences of the MISSION Act on utilization through this analysis, especially due to the timing of MISSION Act implementation shortly before the pandemic began. We did not measure utilization of specialty care services or utilization for patients, so our findings may not be generalizable to other health services. Future work is needed to understand how the utilization of specific types of specialty care changed in the community and VA, especially as the availability of certain types of specialty care is limited in rural areas. We excluded patients who were not assigned to VA primary care during this period from our study; prior studies estimated 89–95% of VA patients were assigned to VA primary care,^17,18^ so our findings may have underestimated the use of community care due to excluded patients. We were also not able to assess the impact of area-level factors such as health professional shortage areas and median household incomes which have been previously shown to be related to the use of community primary care and may be related to the use of mental health and emergency/urgent care in the community as well.^11^

## CONCLUSION

While the VA continued to directly provide many health services, it increased its role as a payer of care after the MISSION Act, which can have significant federal budget and policy implications and implications for patient care. The MISSION Act was associated with substantially higher community care for mental health care and emergency/urgent care with higher take-up in rural areas, in particular. These findings are consistent with access barriers commonly found among Veterans seeking VA mental health and emergency/urgency care, highlighting the challenges and importance of maintaining provider networks in rural areas to ensure access to care for patients in the VA as well as other health care systems. New initiatives focus on providing telehealth services to patients experiencing geographic barriers to care,^19,20^ so better technology can also improve patients’ access to care in rural and other medically underserved areas.

As more services for mental health and emergency/urgent care shift to community providers while the VA continues to provide the vast majority of primary care to Veterans, the increased fragmentation of care across VA and non-VA providers is an area for concern. The need to coordinate care across providers remains paramount. Several review studies have shown that VA care is superior or equivalent to non-VA care for many conditions,^17,18^ so it is critical to track the impact of the MISSION Act on Veterans’ care fragmentation across systems and quality and outcomes of care in the future.

### Supplementary Information

Below is the link to the electronic supplementary material.

Supplementary file1 (DOCX 379 KB)

## Data Availability

Data from this report will not be available to others since data access is restricted under a Memorandum of Understanding between the study team and the VA Office of Primary Care. Statistical code will be made available to others by email request to the corresponding author.
