# Supplementary material for: Growth of Community Outpatient Care in the Veterans Affairs System After the MISSION Act
Source: J Gen Intern Med. 2024 May 9;39(12):2233–40. doi: 10.1007/s11606-024-08787-7 (PMC11347504; doi:10.1007/s11606-024-08787-7)

**Appendix**

Table A1. Test of Quarterly Differences in Total Number of Community Care Visits Before and After the MISSION Act

| Time period | Wald Test P-Value of Quarterly Differences in Mean Number of Clinic Community Care Visits in Each Period versus Pre-MISSION Act Period | | |
| --- | --- | --- | --- |
|  | Primary Care | Mental Health | Emergency/Urgent Care |
| Immediate Post-Mission Act Period | 0.0058 | <0.001 | <0.001 |
| Immediate Post-Covid-19 Emergency Response | 0.4107 | <0.001 | <0.001 |
| Post-COVID-19 Vaccine Distribution | <0.001 | <0.001 | <0.001 |
| Entire Post-MISSION Act Period | <0.001 | <0.001 | <0.001 |

Wald tests were conducted after estimating linear models for number of community care visits adjusting for FY-quarter indicators, clinic characteristics, and rurality.

Table A2. Test of Quarterly Differences in Mean Proportion of Community Care Before and After the MISSION Act

| Time period | Wald Test P-Value of Differences in Adjusted Mean Proportion of Clinic Visits in Each Period versus Pre-MISSION Act Period | | |
| --- | --- | --- | --- |
|  | Primary Care | Mental Health | Emergency/Urgent Care |
| Immediate Post-Mission Act Period | 0.188 | <0.001 | <0.001 |
| Immediate Post-Covid-19 Emergency Response | 0.002 | 0.135 | <0.001 |
| Post-COVID-19 Vaccine Distribution | 0.745 | 0.103 | <0.001 |
| Entire Post-MISSION Act Period | 0.116 | <0.001 | <0.001 |

Wald tests were conducted after estimating probit models for proportion of community care visits adjusting for FY-quarter indicators, clinic characteristics, and rurality.

Figure A1. Mean Number of Visits per Patient per FY-Quarter

Figure A2. Unadjusted proportion of community visits in each quarter, FY2018-2021


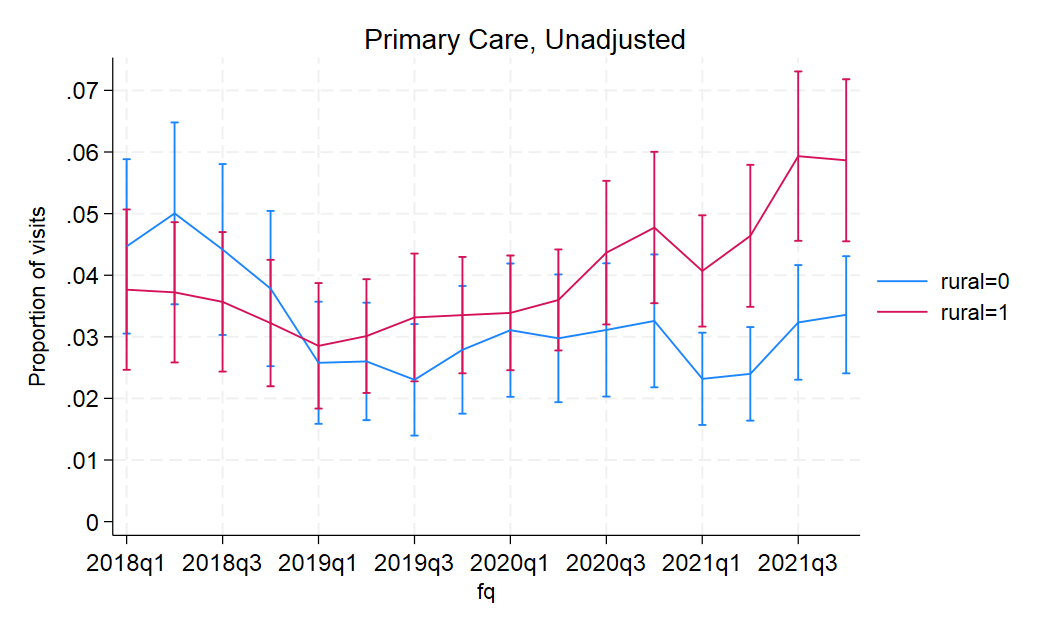

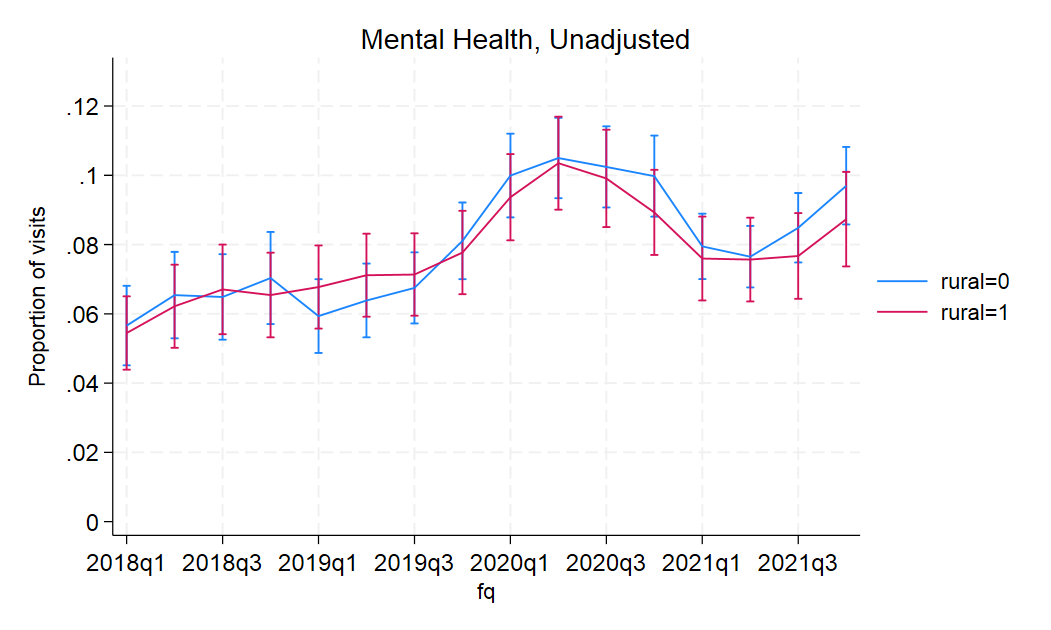

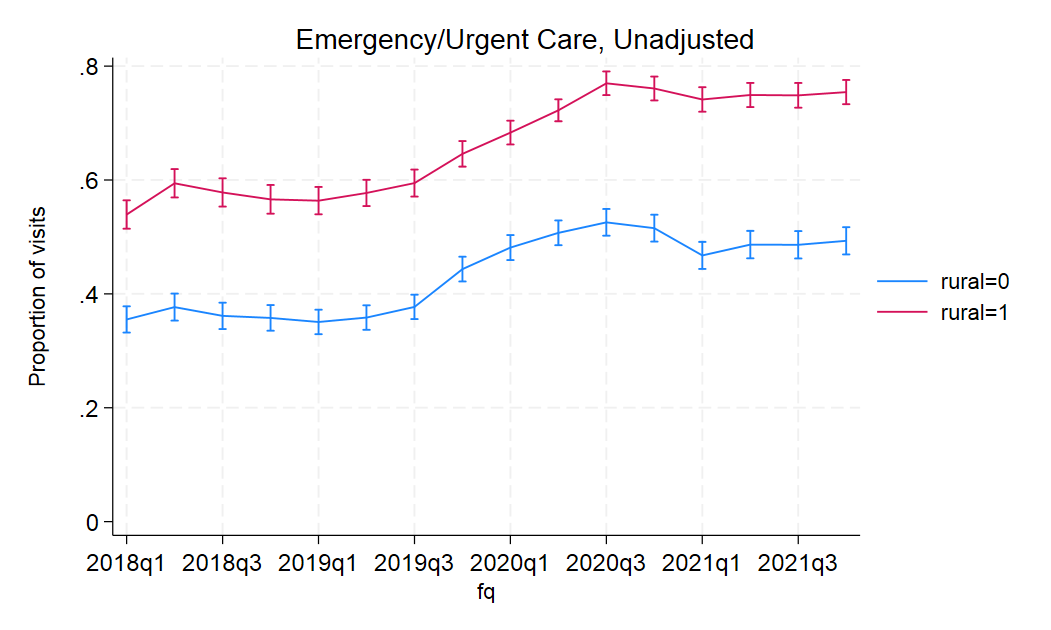


Figure A3. Adjusted Mean Number of Community Care Visits per Site per Quarter, FY2018-2021


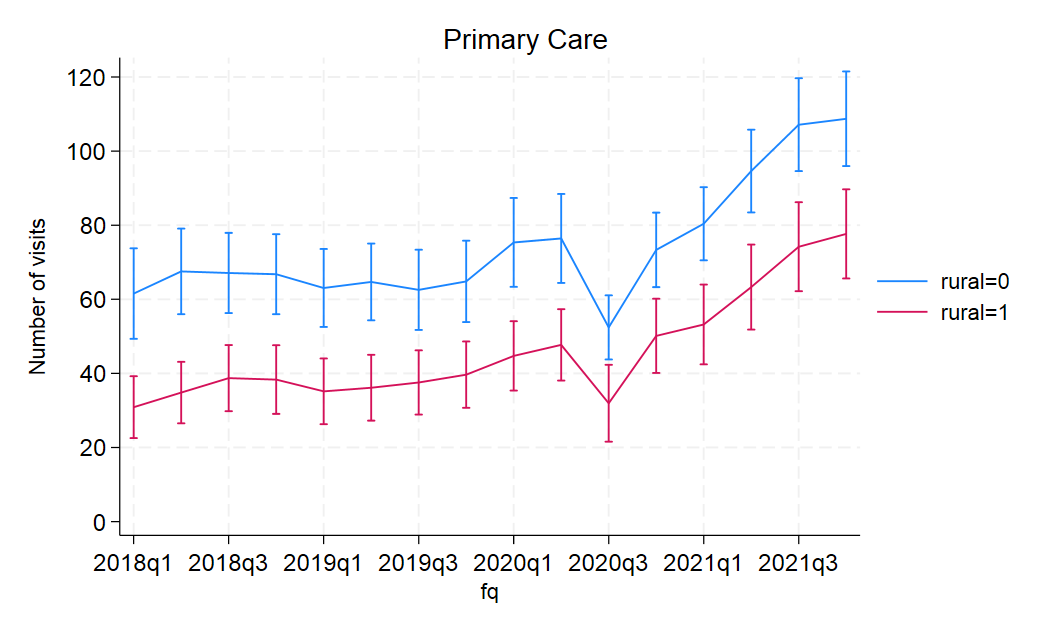

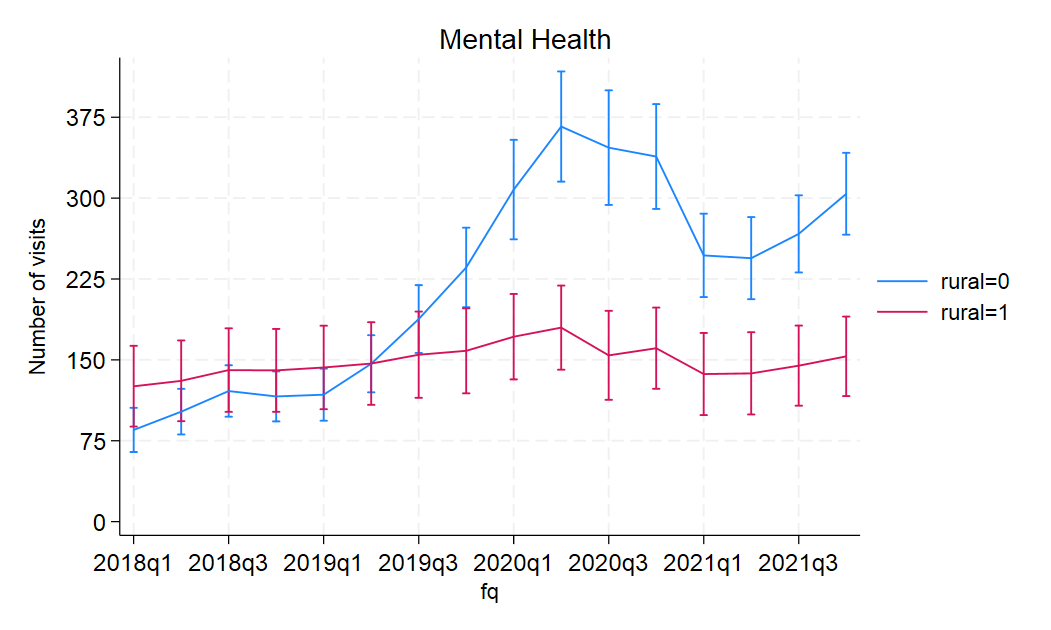

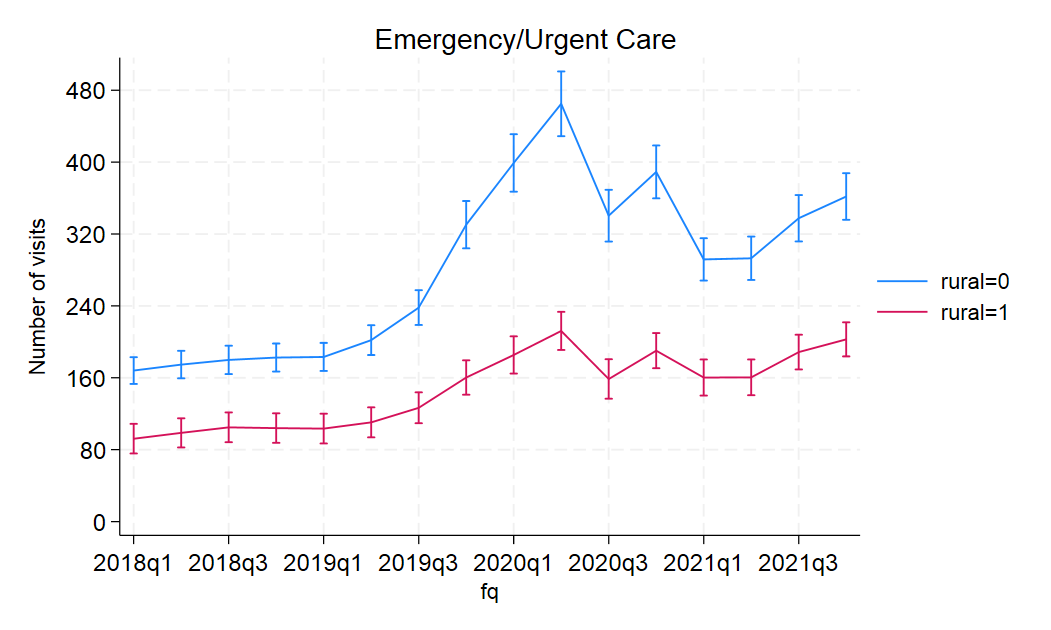

Supplement: Supplementary file 1 — Supplementary file1 (DOCX 379 KB) [file 11606_2024_8787_MOESM1_ESM.docx]
